# Primaquine radical cure in patients with *Plasmodium falciparum* malaria in areas co-endemic for *P falciparum* and *Plasmodium vivax* (PRIMA): a multicentre, open-label, superiority randomised controlled trial

**DOI:** 10.1016/S0140-6736(23)01553-2

**Published:** 2023-12-02

**Authors:** Kamala Thriemer, Tamiru Shibiru Degaga, Michael Christian, Mohammad Shafiul Alam, Megha Rajasekhar, Benedikt Ley, Mohammad Sharif Hossain, Mohammad Golam Kibria, Tedla Teferi Tego, Dagamawie Tadesse Abate, Sophie Weston, Hellen Mnjala, Angela Rumaseb, Ari Winasti Satyagraha, Arkasha Sadhewa, Lydia Vista Panggalo, Lenny L Ekawati, Grant Lee, Rodas Temesgen Anose, Fitsum Getahun Kiros, Julie A Simpson, Amalia Karahalios, Adugna Woyessa, J Kevin Baird, Inge Sutanto, Asrat Hailu, Ric N Price

**Affiliations:** a Global and Tropical Health Division, Menzies School of Health Research and Charles Darwin University, Darwin, NT, Australia; b College of Medicine and Health Sciences, Arba Minch University, Arba Minch, Ethiopia; c Oxford University Clinical Research Unit Indonesia, Jakarta, Indonesia; d International Centre for Diarrhoeal Disease Research, Dhaka, Bangladesh; e Centre for Epidemiology and Biostatistics, Melbourne School of Population and Global Health, University of Melbourne, Melbourne, VIC, Australia; f Arba Minch General Hospital, Arba Minch, Ethiopia; g Eijkman Research Center for Molecular Biology, National Research and Innovation Agency, Cibinong, Indonesia; h Exeins Health Initiative, Jakarta, Indonesia; i Centre for Tropical Medicine and Global Health, Nuffield Department of Medicine, University of Oxford, Oxford, UK; j Ethiopian Public Health Institute, Addis Ababa, Ethiopia; k Department of Parasitology, Faculty of Medicine, University of Indonesia, Jakarta, Indonesia; l College of Health Sciences, Addis Ababa University, Addis Ababa, Ethiopia; m Mahidol-Oxford Tropical Medicine Research Unit, Faculty of Tropical Medicine, Mahidol University, Bangkok, Thailand

## Abstract

**Background:**

In areas co-endemic for *Plasmodium vivax* and *Plasmodium falciparum* there is an increased risk of *P vivax* parasitaemia following *P falciparum* malaria. Radical cure is currently only recommended for patients presenting with *P vivax* malaria. Expanding the indication for radical cure to patients presenting with *P falciparum* malaria could reduce their risk of subsequent *P vivax* parasitaemia.

**Methods:**

We did a multicentre, open-label, superiority randomised controlled trial in five health clinics in Bangladesh, Indonesia, and Ethiopia. In Bangladesh and Indonesia, patients were excluded if they were younger than 1 year, whereas in Ethiopia patients were excluded if they were younger than 18 years. Patients with uncomplicated *P falciparum* monoinfection who had fever or a history of fever in the 48 h preceding clinic visit were eligible for enrolment and were required to have a glucose-6-dehydrogenase (G6PD) activity of 70% or greater. Patients received blood schizontocidal treatment (artemether–lumefantrine in Ethiopia and Bangladesh and dihydroartemisinin–piperaquine in Indonesia) and were randomly assigned (1:1) to receive either high-dose short-course oral primaquine (intervention arm; total dose 7 mg/kg over 7 days) or standard care (standard care arm; single dose oral primaquine of 0·25 mg/kg). Random assignment was done by an independent statistician in blocks of eight by use of sealed envelopes. All randomly assigned and eligible patients were included in the primary and safety analyses. The per-protocol analysis excluded those who did not complete treatment or had substantial protocol violations. The primary endpoint was the incidence risk of *P vivax* parasitaemia on day 63. This trial is registered at ClinicalTrials.gov, NCT03916003.

**Findings:**

Between Aug 18, 2019, and March 14, 2022, a total of 500 patients were enrolled and randomly assigned, and 495 eligible patients were included in the intention-to-treat analysis (246 intervention and 249 control). The incidence risk of *P vivax* parasitaemia at day 63 was 11·0% (95% CI 7·5–15·9) in the standard care arm compared with 2·5% (1·0–5·9) in the intervention arm (hazard ratio 0·20, 95% CI 0·08–0·51; p=0·0009). The effect size differed with blood schizontocidal treatment and site. Routine symptom reporting on day 2 and day 7 were similar between groups. In the first 42 days, there were a total of four primaquine-related adverse events reported in the standard care arm and 26 in the intervention arm; 132 (92%) of all 143 adverse events were mild. There were two serious adverse events in the intervention arm, which were considered unrelated to the study drug. None of the patients developed severe anaemia (defined as haemoglobin <5 g/dL).

**Interpretation:**

In patients with a G6PD activity of 70% or greater, high-dose short-course primaquine was safe and relatively well tolerated and reduced the risk of subsequent *P vivax* parasitaemia within 63 days by five fold. Universal radical cure therefore potentially offers substantial clinical, public health, and operational benefits, but these benefits will vary with endemic setting.

**Funding:**

Australian Academy of Science Regional Collaborations Program, Bill & Melinda Gates Foundation, and National Health and Medical Research Council.

## Introduction

*Plasmodium vivax* malaria is becoming the predominant cause of malaria in many regions, accounting for 4–7 million annual cases in 49 endemic countries in Asia, Oceania, the Horn of Africa, and the Americas.^1^ The control and elimination of *P vivax* is confounded by the parasite's ability to form dormant liver stages (ie, hypnozoites) that can reactivate weeks or months after an acute infection (ie, relapse). Relapses are associated with a febrile illness and a cumulative risk of direct and indirect morbidity and mortality^2^, ^3^, ^4^, ^5^ and are an important source of onward transmission of the parasite.^6^, ^7^, ^8^

**Research in context**

**Evidence before this study**

Radical cure with primaquine or tafenoquine is currently only recommended in patients presenting with *Plasmodium vivax* malaria. Before inception of the study a systematic literature review and meta-analysis was done using MEDLINE, Embase, Web of Science, and the Cochrane Database of Systematic Reviews to identify prospective clinical trials in any language, published between Jan 1, 1960, and Jan 5, 2018, that assessed drug efficacy in patients with uncomplicated *Plasmodium falciparum* malaria in countries co-endemic for *P vivax*. The search identified 153 *P falciparum* efficacy studies enrolling 31 262 patients and showed a high risk of *P vivax* parasitaemia after treatment of *P falciparum* malaria, particularly in areas with short relapse periodicity and after treatment with artemisinin-based combination therapies that are rapidly eliminated. A 2020 individual patient data meta-analysis of 15 341 patients with *P falciparum* confirmed these findings. Prospective trials are required to explore the risks and benefits of providing radical cure to patients with either *P vivax* or *P falciparum* (universal radical cure) in different endemic settings.

**Added value of this study**

This multicentre trial assessed the safety and efficacy of high-dose short-course primaquine radical cure in patients presenting with uncomplicated *P falciparum* malaria to prevent subsequent *P vivax* recurrences. The study showed that, in patients who have a glucose-6-dehydrogenase (ie, G6PD) activity of 70% or greater, this regimen was safe and led to a significant reduction in the risk of subsequent *P vivax* parasitaemia.

**Implications of all the available evidence**

Expanding the current indication for radical cure to include patients presenting with *P falciparum* has substantial clinical, public health, and operational benefits, but these benefits will vary with endemic setting.

In areas co-endemic for *P vivax* and *Plasmodium falciparum* there is an increased risk of *P vivax* parasitaemia following falciparum malaria, substantially higher than would be expected from the risk of re-infection alone.^9^, ^10^, ^11^, ^12^, ^13^ Patients presenting with acute falciparum malaria are also likely to have had previous *P vivax* infection^14^ and at the time of presentation could have either undetected low level parasitaemia or hypnozoites. Both fever and haemolysis associated with malaria have been hypothesised to stimulate the reactivation of hypnozoites resulting in subsequent recurrent episodes of malaria.^15^, ^16^, ^17^

Pooled analyses suggest that the greatest risk of *P vivax* parasitaemia following *P falciparum* is in regions of short relapse periodicity and following treatment with rapidly eliminated artemisinin-based combination therapy, such as artemether–lumefantrine.^10^, ^11^ Since a high proportion of *P vivax* parasitaemia infections have peripheral parasitaemia with sexual stages^18^ these relapses have the potential to sustain ongoing parasite transmission. Collectively, these findings suggest that in co-endemic regions there is a rationale for opportunistically eradicating *P vivax* hypnozoites from the liver in patients presenting with uncomplicated *P falciparum* malaria, an approach termed universal radical cure.

Most endemic countries currently recommend a low-dose regimen of primaquine (total dose 3·5 mg/kg) for the radical cure of *P vivax* malaria, administered over 14 days. This prolonged treatment limits the daily dose of primaquine to 0·25 mg/kg per day to improve tolerability and reduce the risk of drug-induced haemolysis. However, extended drug regimens with treatment for more than 10 days after resolution of acute symptoms are associated with poor adherence and effectiveness.^19^, ^20^ Several studies have assessed short-course regimens in the expectation that these will improve adherence and therefore effectiveness. In 2022, WHO endorsed a 7-day regimen (0·5 mg/kg per day, total dose 3·5 mg/kg primaquine), which has been widely used for more than a decade in South America.^21^ However, higher total doses of primaquine offer even greater antirelapse efficacy, and this benefit is apparent in most endemic areas.^22^ A higher dose regimen (7 mg/kg total dose) given over 7 days (1·0 mg/kg per day) has been shown to be non-inferior to the same total dose given over 14 days (0·5 mg/kg per day),^23^ and newly developed point-of-care tests for glucose-6-dehydrogenase (G6PD) deficiency are now available to guide safer treatment.^24^ The potential to provide radical cure safely with a short-course high-dose regimen of primaquine provides major practical advantages and extending the indication for radical cure to other patient groups at high risk of recurrent *P vivax* has the potential to affect vivax elimination.

To better understand the risks and benefits of universal radical cure, we did a multicentre randomised, open-label trial to assess the safety of a high-dose short-course primaquine treatment (total dose 7 mg/kg over 7 days) in patients with 70% G6PD activity or greater and *P falciparum* malaria. The trial also aimed to assess the efficicacy of universal radical cure to reduce the risk of subsequent *P vivax* episodes compared with the current standard treatment for *P falciparum*, which entails schizontocidal treatment of blood stage parasites plus a single dose of primaquine to eliminate the sexual parasite stages.

## Methods

### Study design

We did a multicentre, open-label, superiority randomised controlled trial to assess short-course high-dose primaquine treatment to prevent *P vivax* parasitaemia in patients with 70% G6PD activity or greater, who were presenting with uncomplicated *P falciparum* monoinfection. The study was done in one health clinic in Bangladesh (in Alikadam), three in Indonesia (one each in Mangili, Waijeli, and Tanaraing), and one in Ethiopia (in Arba Minch; appendix p 2). The detailed study protocol has been published previously.^25^ This study was approved by the NT Health and Menzies School of Health Research Human Research Ethics Committee and by the respective independent review boards in the participating study sites (appendix p 4).

### Participants

Patients presenting with uncomplicated *P falciparum* monoinfection who had fever or a history of fever in the 48 h preceding clinic visit were eligible for enrolment. Individuals were required to have a G6PD activity of 70% or greater of the adjusted male median of the study population measured using the STANDARD G6PD (SD Biosensor, Gyeonggi-do, South Korea) for inclusion. Infants younger than 1 year were excluded in Bangladesh and Indonesia, and individuals younger than 18 years were excluded in Ethiopia. Patients were also excluded if they had signs or symptoms of severe malaria, a haemoglobin concentration below 8 g/dL, were pregnant or breastfeeding, had any known hypersensitivity to the study drugs, were regularly using other drugs with haemolytic potential, or had a transfusion within the last 4 months. Before enrolment, written informed consent was obtained from the patient or their guardian. Written assent was also obtained from patients who were aged 11–18 years.

### Randomisation and masking

Eligible patients were treated with a blood schizontocidal drug and were randomly assigned in a 1:1 ratio to either receive high-dose short-course primaquine (1 mg/kg per day for 7 days) or standard care (single dose primaquine as transmission blocking agent). The allocation sequence was done by an independent statistician in blocks of eight. Sealed envelopes containing allocations were prepared by an independent team member before study start. Individual envelopes were only opened, thereby allocating patients to a treatment group, after the screening was completed for the patients who were enrolled into the study. All treatments were open label. Clinicians at the health clinics were responsible for enrolling participants.

### Procedures

All patients were treated as outpatients with the local first-line treatment for *P falciparum* according to national guidelines: artemether–lumefantrine in Ethiopia and Bangladesh and dihydroartemisinin–piperaquine in Indonesia.

Patients in the intervention arm received 7 days of oral high-dose primaquine (1 mg/kg per day; appendix p 7). These patients were asked to return to the health facility daily on days 1–6 for directly supervised treatment of schizontocidal and primaquine treatment. For patients treated with artemether–lumefantrine, only the morning dose was supervised, and tablets were given for self-administration of the evening dose at home.

Patients in the standard care arm received a single dose of oral primaquine (0·25 mg/kg) to reduce the risk of falciparum transmission, as per national guidelines (appendix p 8), which was administered with their schizontocidal treatment and supervised in the same way as for patients in the intervention arm.

At enrolment, a medical history was taken, and a physical examination done. After completion of treatment, patients were followed up weekly from day 7 until day 63. At each visit (including visits on days 3–6 in the intervention arm), a medical history was taken by study clinicians, a symptom questionnaire completed, and any adverse events or serious adverse events were recorded (appendix p 8). Patients were encouraged to report to the study centre if they became unwell. Patients who missed their scheduled follow-up visits were contacted either in person or by telephone by study staff and encouraged to return to the study centre for review or were visited at home.

### Outcomes

The primary endpoint was the incidence risk of *P vivax* parasitaemia (symptomatic or asymptomatic) by day 63. Secondary outcomes included the incidence risks of symptomatic *P vivax* parasitaemia at day 63; any *P vivax* parasitaemia at day 28 and day 42; any *P falciparum* parasitaemia at day 28, day 42, and day 63; and *P falciparum* gametocytaemia between day 7 and day 63. Further secondary endpoints included parasite clearance and fever clearance.

There were multiple safety outcomes. First the proportion of patients vomiting their medication on the day of enrolment within 1 h of administration and the proportion of patients vomiting any of their primaquine doses within 1 h of administration. Second, the proportion of adverse events and serious adverse events, as well as the proportion of patients experiencing severe (haemoglobin <5 g/dL) and moderately severe (haemoglobin <7 g/dL) anaemia or the risk for blood transfusion between day 2 and day 7. Finally, the proportion of patients experiencing a 25% or greater reduction in haemoglobin to under 7 g/dL with and without haemoglobinuria at day 2 and day 7, which was defined as a clinically significant event associated with increased mortality.^3^

### Statistical analysis

The analysis was conducted according to an a priori statistical analysis plan.^25^

The study was powered assuming that the risk of *P vivax* infection after *P falciparum* is highest when treated with artemether–lumefantrine, used in Bangladesh and Ethiopia. Assuming a risk of 41% for *P vivax* after *P falciparum* malaria at day 63 after artemether–lumefantrine treatment, and a reduction of this risk to 20% in the intervention arm,^9^, ^10^, ^11^ a total sample size of 322 patients was calculated to have 98% power at the two-sided 5% significance level. Assuming a loss to follow-up rate of 20%, the sample size was increased to 403 across the sites in Ethiopia and Bangladesh where artemether–lumefantrine is used. A further 100 patients were recruited in Indonesia and treated with dihydroartemisinin–piperaquine. We planned to pool data from all sites if site-specific differences were not observed or could not be calculated, which would result in more than 99% power for the primary outcome and additional power for the secondary outcomes.

For the primary efficacy outcome, cumulative incidence risks of *P vivax* parasitaemia between day 7 and day 63 in the standard care arm and 7-day primaquine arm were estimated by Kaplan-Meier analysis and hazard ratios (HRs; 95% CI) were estimated by Cox regression for time to first recurrence. HRs (95% CI) were also estimated using Cox regression for the secondary efficacy outcomes of *P vivax* parasitaemia on days 28 and 42; *P falciparum* parasitaemia on day 28, day 42, and day 63; and *P falciparum* gametocytaemia between day 7 and day 63. Subgroup analyses for the primary outcomes were defined a priori for schizontocidal treatment and post hoc by site. The main analysis was done on the intention-to-treat population. For the per-protocol analysis, a causal diagram was created to identify pre-randomisation and post-randomisation confounders (appendix p 9).

The intention-to-treat population was defined as all eligible patients who were randomly assigned to study groups. In the per-protocol analysis, patients who had substantial protocol violations (appendix p 10) or did not complete treatment were excluded. The safety analysis included all eligible patients who were randomly assigned to a treatment group and received any study drug.

All analyses were performed using Stata version 17.0.

### Role of the funding source

The funders of the study had no role in study design, data collection, data analysis, data interpretation, or writing of the report.

## Results

**Figure 1:**
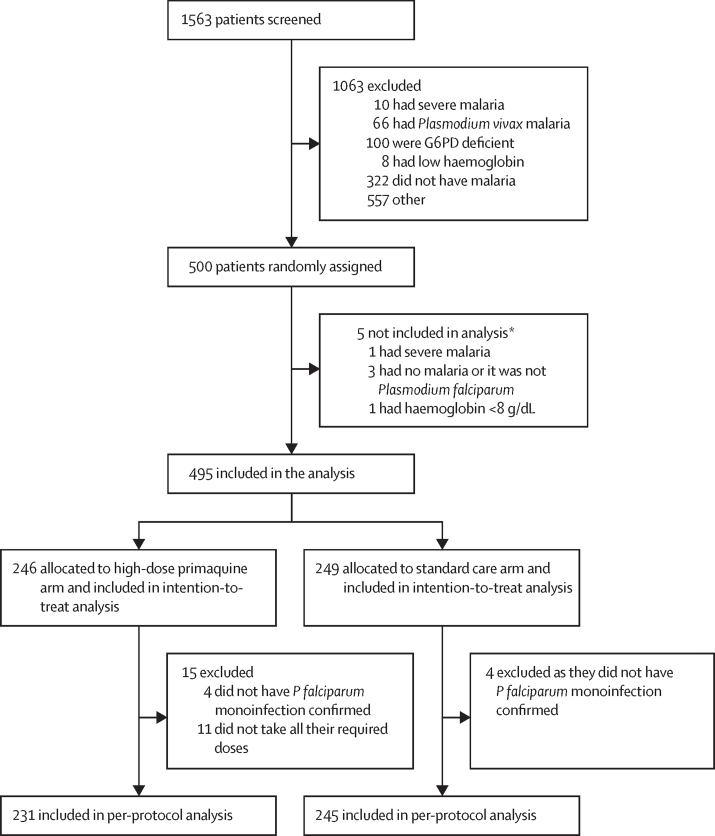
CONSORT diagram G6PD=glucose-6-dehydrogenase. *Of the five patients randomly assigned but not included in the analysis, three were assigned to the high-dose primaquine arm and two to the standard care arm.

Between Aug 18, 2019, and March 14, 2022, a total of 1563 patients were screened for inclusion. Due to the COVID-19 pandemic the study was interrupted after enrolment was completed in Bangladesh but before enrolment could start in Ethiopia and Indonesia (appendix p 10). A total of 1063 (68%) patients did not meet the enrolment criteria and 500 patients were enrolled into the study, of whom five were subsequently excluded since they did not fulfil the eligibility criteria (figure 1). 495 patients were included in the intention-to-treat analysis, 246 in the intervention arm and 249 in the standard care arm. Baseline patient characteristics were similar between the two study groups (table 1). A high proportion of patients were adults (87%), reflecting exclusion of children in Ethiopia. The median *P falciparum* parasitaemia was 11 920 parasites per μL (IQR 4500–32 400) in the intervention arm and 16 600 parasites per μL (5500–34 600) in the standard care arm. The mean total dose of primaquine given in the intervention arm was 7·2 mg/kg (range 0·9–9·1). All patients completed schizontocidal treatment and 234 (95%) of the 246 patients randomly assigned to the intervention arm had all seven primaquine doses. In the intervention arm, 99% (1660 of 1667) of primaquine doses were provided with food (eg, cracker, biscuits, or banana). One patient in the intervention arm (on day 2) and two in the standard care arm (both on day 0) vomited their medication within 1 h of administration, but tolerated re-administration of medication.

**Table 1:** Baseline characteristics

		High-dose primaquine*(n=246)	**Standard care**†**(n=249)**
Site			
	Bangladesh	23 (9%)	24 (10%)
	Indonesia	48 (20%)	50 (20%)
	Ethiopia	175 (71%)	175 (70%)
Age			
	Median (IQR), years	22·0 (18·0–30·0)	21·2 (18·0–30·0)
	6 months to <12 months	0	1 (<1%)
	12 months to <5 years	6 (2%)	2 (1%)
	5 years to <15 years	21 (9%)	34 (14%)
	≥15 years	219 (89%)	212 (85%)
Sex			
	Male	153 (62%)	159 (64%)
	Female	93 (38%)	90 (36%)
Bodyweight			
	Median (IQR), kg	54 (45–61)	54 (46–60)
	9 kg to <18 kg	9 (4%)	10 (4%)
	18 kg to <36 kg	15 (6%)	26 (10%)
	≥36 kg	222 (90%)	213 (86%)
*P falciparum* parasites per μL		11 920 (4500–32 400)	16 600 (5500–34 600)
Gametocyte carriage at enrolment		24 (10%)	22 (9%)
*P falciparum* gametocytes per μL		48 (31–741)	300 (64–488)
Body temperature, °C		37·5 (1·0)	37·4 (1·1)
Fever‡		116 (47%)	108 (43%)
Haemoglobin, g/dL			
	Mean (SD)	14·2 (2·5)	14·0 (2·5)
	Range	8·4–21·9	8·1–24·1
G6PD activity, U/gHb		7·2 (6·3–8·1)	7·0 (6·3–8·1)

Data are n (%), median (IQR), or mean (SD), unless otherwise specified. G6PD=glucose-6-dehydrogenase. *P falciparum*=*Plasmodium falciparum*.

* Schizontocidal treatment of blood stage parasites plus 7 mg/kg total dose of primaquine given over 7 days.

† Schizontocidal treatment of blood stage parasites plus a single dose of primaquine to elimate the sexual parasite stages.

‡ Defined as axillary temperature of ≥37·5°C.

**Figure 2:**
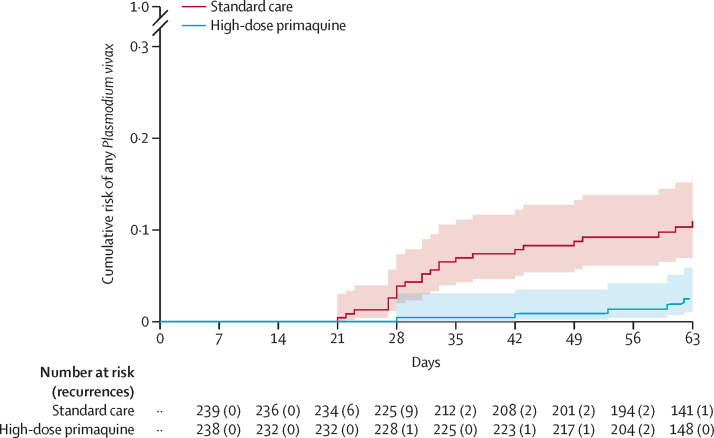
Risk of *Plasmodium vivax* after *Plasmodium falciparum* malaria until day 63 Kaplan-Meier graph showing the risk of any *P vivax* malaria at day 63 in patients enrolled with *P falciparum* malaria infection who were treated with a 7-day course of high-dose primaquine versus standard care. Shading shows 95% CI.

**Figure 3:**
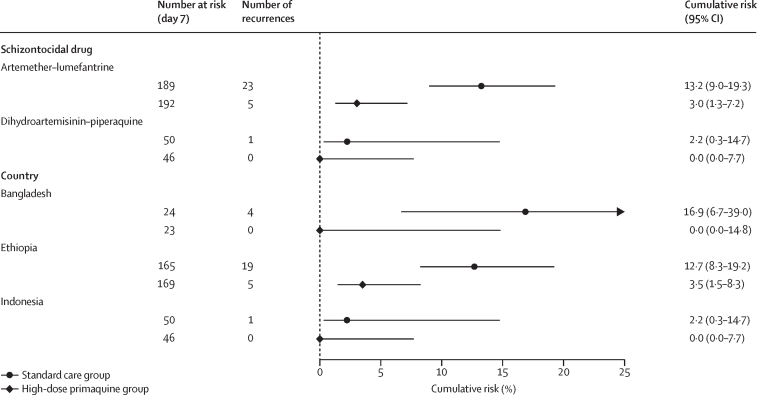
Risk of *P vivax* after *P falciparum* malaria at day 63 by schizontocidal drug and study site Artemether–lumefantrine was used in Bangladesh and Ethiopia and dihydroartemisinin–piperaquine was used in Indonesia. For subgroups where no outcome events were observed, the 95% CI was estimated as a proportion of N at risk on day 7 using the binomial exact method.

At day 63, the incidence risk of any *P vivax* parasitaemia was 11·0% (95% CI 7·5–15·9) in the standard care arm compared with 2·5% (1·0–5·9) in the intervention arm (HR 0·20, 95% CI 0·08–0·51; p=0·0009; figure 2, table 2). The difference in incidence risk between treatment arms varied by schizontocidal treatment and by site. In Indonesia, where patients were treated with dihydroartemisinin–piperaquine, the risk of *P vivax* parasitaemia was 2·2% (95% CI 0·3–14·7) in the standard care arm and 0·0% (0·0–7·7) in the intervention arm, whereas in Bangladesh and Ethiopia where patients were treated with artemether–lumefantrine the corresponding risk in the standard care arm was 13·2% (9·0–19·3) and in the intervention arm was 3·0% (1·3–7·2; HR 0·20, 95% CI 0·08–0·54; p=0·0013; figure 3; appendix pp 11–12).

**Table 2:** Efficacy outcomes

	**Incidence risk (95% CI)**		**HR (95% CI)**	**p value**
	High-dose primaquine*	Standard care†		
**Day 63**				
Any *Plasmodium vivax* parasitaemia	2·5 (1·0–5·9)	11·0 (7·5–15·9)	0·20 (0·08–0·51)	0·0009
Symptomatic *P vivax* parasitaemia	0·4 (0·1–3·1)	4·0 (2·0–8·0)	0·12 (0·01–0·92)	0·042
Any *Plasmodium falciparum* malaria	7·6 (4·8–12·0)	8·2 (5·1–13·1)	1·01 (0·51–2·00)	0·98
*P falciparum* gametocytaemia	0·0 (0·0–1·5)‡	1·0 (0·2–3·8)	..§	..§
**Day 42**				
Any *P vivax* malaria	1·0 (0·2–3·9)	8·0 (5·1–12·5)	0·11 (0·03–0·46)	0·0026
Any *P falciparum* malaria	1·9 (0·7–4·9)	4·0 (2·0–7·8)	0·49 (0·15–1·64)	0·25
**Day 28**				
Any *P vivax* malaria	0·5 (0·1–3·7)	4·4 (2·3–8·2)	0·11 (0·01–0·90)	0·039
Any *P falciparum* malaria	1·0 (0·2–3·8)	2·0 (0·7–5·2)	0·49 (0·09–2·70)	0·42

Data are median (IQR). HR=hazard ratio.

* Schizontocidal treatment of blood stage parasites plus 7 mg/kg total dose of primaquine given over 7 days.

† Schizontocidal treatment of blood stage parasites plus a single dose of primaquine to elimate the sexual parasite stages.

‡ 95% CI estimated as a proportion of N at risk on day 7 using the binomial exact method.

§ Could not be estimated due to zero events in the intervention arm.

The incidence risk of symptomatic *P vivax* parasitaemia at day 63 was also significantly lower in the intervention arm (0·4%, 95% CI 0·1–3 ·1) compared with the standard care arm (4·0%, 2·0–8·0; HR 0·12, 95% CI 0·01–0·92; p=0·042). There was no difference in the risk of *P falciparum* parasitaemia between treatment arms (1·01, 95% CI 0·51–2·00; p=0·98; table 2). Results were similar in the per-protocol analysis (appendix p 13).

At enrolment, *P falciparum* gametocytes were present in 22 (9%) of the 249 patients in the standard care arm and 24 (10%) of the 246 in the intervention arm. Of the 46 patients presenting with *P falciparum* gametocytes, no patients were gametocytaemic on day 7. Overall, the risk of *P falciparum* gametocyte carriage during follow-up was 1% (95% CI 0·2–3·8) in the standard care arm and 0% (0·0–1·5) in the intervention arm.

Fever clearance was similar between groups with 98 (91%) of 108 patients becoming afebrile within 24 h of starting treatment in the standard care arm compared with 96 (84%) of 114 in the intervention arm (p=0·14). Overall, 454 (93%) of 487 patients with peripheral parasitaemia had complete parasite clearance within 2 days of starting treatment, with no significant difference between treatment groups (p=0·96; appendix p 13).

A routine symptom checklist was done on day 2 and day 7 in both treatment arms. Gastrointestinal tolerability was similar between arms. In the standard care arm 16 (7%) of 237 patients reported vomiting, seven (3%) of 236 reported diarrhoea, and 92 (39%) of 237 reported loss of appetite on day 2. The corresponding proportions in the intervention arm were 6% (15 of 235), 2% (4 of 235), and 37% (88 of 235). On day 7 gastrointestinal intolerance was low and similar between both treatment arms (table 3).

**Table 3:** Safety and tolerability outcomes

	High-dose primaquine*	**Standard care**†
**Adverse events until day 42**		
All adverse events	97/246 (39%)	46/249 (19%)
Primaquine related‡	26/246 (11%)	4/249 (2%)
Primaquine unrelated‡	71/246 (29%)	42/249 (17%)
Grade 1	90/246 (37%)	42/249 (17%)
Grade 2	6/246 (2%)	4/249 (2%)
Grade 3	1/246 (<1%)	0/249
**Adverse events for days 0–7**§		
All adverse events	54/246 (22%)	17/249 (7%)
Primaquine related‡	20/246 (8%)	3/249 (1%)
Primaquine unrelated‡	34/246 (14%)	14/249 (6%)
**Adverse events for days 8–42**		
All adverse events	43/246 (18%)	29/249 (12%)
Primaquine related‡	6/246 (2%)	1/249 (<1%)
Primaquine unrelated‡	37/246 (15%)	28/249 (11%)
**Serious adverse events until day 63**		
Primaquine related‡	1/246 (<1%)	0/249
Primaquine unrelated‡	1/246 (<1%)	0/249
**Vomiting within 1 h of treatment administration**¶		
Any medication	1/1689 (<1%)	2/791 (<1%)
Any primaquine dose	1/1689 (<1%)	1/248 (<1%)
**Symptoms reported for day 2**‖		
Vomiting	15/235 (6%)	16/237 (7%)
Headache	96/235 (41%)	117/237 (49%)
Nausea	31/235 (13%)	28/237 (12%)
Diarrhoea	4/235 (2%)	7/236 (3%)
Loss of appetite	88/235 (37%)	92/237 (39%)
Abdominal pain	29/235 (12%)	24/236 (10%)
Muscle pain	87/235 (37%)	92/237 (39%)
Joint pain	84/235 (36%)	91/237 (38%)
Fever	72/235 (31%)	88/237 (37%)
Dark urine	21/235 (9%)	22/237 (9%)
Dizziness	72/235 (31%)	79/237 (33%)
Shortness of breath	0/235	3/235 (1%)
Irritability	13/235 (6%)	17/237 (7%)
Jaundice	0/235	0/237
Fatigue	93/235 (40%)	104/237 (44%)
Malaise	62/235 (26%)	84/237 (35%)
Chills	46/235 (20%)	68/237 (29%)
**Symptoms reported for day 7**‖		
Vomiting	4/193 (2%)	1/186 (1%)
Headache	9/193 (5%)	15/187 (8%)
Nausea	4/193 (2%)	4/186 (2%)
Diarrhoea	3/193 (2%)	1/187 (1%)
Loss of appetite	7/192 (4%)	11/187 (6%)
Abdominal pain	18/193 (9%)	6/187 (3%)
Muscle pain	3/193 (2%)	7/186 (4%)
Joint pain	3/193 (2%)	6/187 (3%)
Fever	8/193 (4%)	10/187 (5%)
Dark urine	6/193 (3%)	11/187 (6%)
Dizziness	5/192 (3%)	9/187 (5%)
Shortness of breath	0/192	0/187
Irritability	0/193	5/187 (3%)
Jaundice	1/193 (1%)	0/187
Fatigue	3/193 (2%)	5/187 (3%)
Malaise	4/193 (2%)	7/187 (4%)
Chills	2/193 (1%)	5/187 (3%)
**Patients with anaemia on day 2**		
Moderately severe (haemoglobin between 5 g/dL and 7 g/dL)	1/104 (1%)	1/109 (1%)
Severe (haemoglobin less than 5 g/dL)	0/104	0/109
**Patients with anaemia on day 7**		
Moderately severe (haemoglobin between 5 g/dL and 7 g/dL)	2/197 (1%)	2/194 (1%)
Severe (haemoglobin less than 5 g/dL)	0/197	0/194

* Schizontocidal treatment of blood stage parasites plus 7 mg/kg total dose of primaquine given over 7 days.

† Schizontocidal treatment of blood stage parasites plus a single dose of primaquine to elimate the sexual parasite stages.

‡ Related events include those that are possibly, probably, or definitely related.

§ Reported retrospectively on day 7 for patients in the standard care arm.

¶ Denominators are total number of doses.

‖ Denominators vary based on availability of data.

A total of 143 adverse events were reported between enrolment and day 42, of which 132 (92%) were mild (grade 1; table 3). Reporting of adverse events varied considerably by site with a low number of events reported in Bangladesh, and higher numbers in Indonesia (appendix pp 14–18).

71 (50%) of the 143 adverse events were reported before or on day 7 (54 in the intervention arm and 17 in the standard care arm). Patients in the intervention arm had scheduled visits on days 3–6 in which adverse events were assessed; 29 (54%) of the reported events for this group occurred on those visit days. Patients in the standard care arm did not have scheduled visits on days 3–6 and reported events retrospectively at their day 7 visit; two (12%) of the 17 events reported for this group occurred during days 3–6 (appendix p 20). There were only four adverse events reported to be related to primaquine in the standard care arm and 26 in the intervention arm (table 3; appendix p 21) between day 0 and day 42. 23 (77%) of the 30 related events occurred before or on day 7.

One adverse event was reported as grade 3 occurring in a patient enrolled in the intervention arm. The patient was an 18-year-old male presenting with acute gastritis and vomiting on day 5, treated as an outpatient with an antiemetic. He continued his primaquine course and recovered within 5 days.

There were two serious adverse events reported, both of which occurred in the intervention arm. The first event was in a male patient aged 24 years who was admitted to hospital with severe abdominal pain on day 7, after completing primaquine treatment. He had been taking non-steroidal, anti-inflammatory drugs for several years for myalgia after physical activity. The event was considered to be possibly related to the study drug compounded by underlying gastritis. The patient made a rapid recovery and was discharged the following day. The second event was in a male patient aged 45 years who presented with abdominal pain on day 25 and signs of sepsis, both of which were considered to be unrelated to the study drug. He was treated with ciprofloxacin, analgesia, and intravenous fluids in hospital. He made a rapid recovery and was discharged the following day.

The mean haemoglobin concentration at enrolment was 14·1 g/dL (SD 2·5, range 8·1 to 24·2) and was similar between treatment arms. The mean haemoglobin fell to 12·6 g/dL in the intervention arm and to 12·7 g/dL in the standard care arm on day 2 and was 13·5 g/dL in the intervention arm and 13·9 g/dL in the standard care arm on day 7. On day 2 the unadjusted mean absolute change in haemoglobin relative to baseline was –0·5 g/dL (range –4·6 to –4·4) in the standard care arm and –0·3 g/dL (–9·2 to 5·7) in the intervention arm. By day 7, the unadjusted mean absolute change in haemoglobin relative to baseline was –0·1 g/dL (–5·6 to 6·4) in the standard care arm and –0·7 g/dL (–6·9 to 8·7) in the intervention arm (appendix p 23).

None of the patients developed severe anaemia (haemoglobin of <5 g/dL; table 3). By day 7, two patients in the standard care arm and two patients in the intervention arm had a reduction in haemoglobin of 25% or more and had developed moderately severe anaemia (<7 g/dL). Three of these patients (two in the intervention arm and one in the standard care arm) reported haemoglobinuria. Only one of these patients (in the standard care arm) was symptomatic and reported dizziness but no shortness of breath. All patients continued their treatment without interruption, no patient required a blood transfusion, and they all recovered fully by day 63 (appendix p 24).

## Discussion

This multicentre, open-label, superiority randomised trial provides prospective evidence of the efficacy of universal radical cure across different epidemiological settings. In patients presenting with uncomplicated falciparum malaria, high-dose short-course primaquine treatment (total dose 7 mg/kg over 7 days) was safe and relatively well tolerated and reduced the risk of subsequent *P vivax* parasitaemia within 63 days by five-fold. Radical cure is likely to continue to offer protection after day 63 meaning that these efficacy estimates are probably conservative.

However, there was marked heterogeneity between sites. In the standard care arm, the risk of *P vivax* parasitaemia during follow-up was 12·7% in Ethiopia, 16·9% in Bangladesh, and 2·2% in Indonesia. This heterogeneity might reflect the prolonged post-treatment prophylaxis afforded by the slowly eliminated dihydroartemisinin–piperaquine, which is used for blood stage activity in Indonesia, compared with artemether–lumefantrine, which was used in Ethiopia and Bangladesh. The corresponding absolute reduction in the risk of *P vivax* following high-dose primaquine was 10·3% following artemether–lumefantrine, but only 2·3% following dihydroartemisinin–piperaquine. Alternatively, the site differences could be explained by differing transmission intensity and corresponding hypnozoite burden, which are particularly low in the Indonesian study site.

The absolute risks of *P vivax* parasitaemia following *P falciparum* were also considerably lower than expected from a previous pooled analysis,^11^ in which a risk of nearly 40% was reported in patients. This result is probably a reflection of the decreasing burden of malaria at all three sites, resulting in a reduction of the risk of *P vivax* infection after *P falciparum* infection. A longitudinal analysis of patients from the Thailand–Myanmar border from 2003 to 2010 showed that the risk of *P vivax* recurrence after initial *P falciparum* infection fell from more than 20% in 2003 to less than 5% in 2010.^12^ Universal radical cure is therefore likely to have a greater effect on *P vivax* recurrences in highly endemic areas and areas with high relapse periodicity. Evidence from a cluster-randomised trial^13^ comparing supervised versus unsupervised provision of universal radical cure in Papua, Indonesia, where malaria is highly endemic, showed more than a 60% reduction in the risk of *P vivax* 6 months after supervised radical cure treatment compared with unsupervised radical cure.

The most recent WHO antimalarial treatment guidelines include a recommendation against high-dose short-course primaquine, citing concerns over serious adverse events at this higher daily dosage including gastrointestinal tolerability.^21^ Increased gastrointestinal symptoms have been reported with higher daily doses of primaquine in previous trials,^23^, ^26^ but tolerability can be mitigated by ingestion of primaquine with food.^27^ Patients in our trial were explicitly advised to take their medication with food such as crackers, biscuits, or bananas and these were provided by study staff. All but seven of the 1667 primaquine doses in the intervention arm were taken with food and were generally well tolerated. Based on the symptom checklist done on day 2 and day 7, gastrointestinal symptoms, such as loss of appetite, occurred in nearly 40% of patients and this result was similar between patients receiving high daily doses of primaquine in the intervention arm and single low-dose primaquine in the standard care arm. However, there were a greater number of adverse events reported in the intervention group. The proportional increase of adverse events between treatment arms mainly arose on days 3–6 and these events were generally mild (ie, grade 1). During this period, patients in the intervention arm were seen for supervised treatment, whereas those in the standard care arm reported events for those days in retrospect on day 7, meaning that the standard care arm might be confounded by recall bias.

Although co-administration of high-dose primaquine with food is likely to have mitigated the risks of gastrointestinal intolerability, one of the two serious adverse events occurred after the last day of treatment, requiring hospital admission. The event occurred 1 day after completion of treatment and was attributed to underlying chronic gastritis, although this could have been exacerbated by high-dose primaquine. Wider scale roll-out of high-dose primaquine should include caution in patients with premorbid gastrointestinal conditions.

The greatest concern for the introduction of shorter more effective radical cure options is that of severe haemolysis in individuals with low G6PD activity.^28^ In our study, patients were only enrolled if they had enzyme activity of 70% or greater. This conservative cut-off was chosen because the risk–benefit ratio of a high primaquine dose is likely to be different in this patient population compared with patients who receive radical cure traditionally. Reassuringly, in patients with a G6PD activity of 70% or greater, high-dose primaquine was well tolerated with no reported haemolytic adverse events. The reduction in haemoglobin was similar between groups, and was probably driven by the acute falciparum parasitaemia rather than the drug.^29^

There are substantial logistical challenges in delivering malaria radical cure safely and effectively.^28^ A novel quantitative G6PD testing option (STANDARD G6PD, SD Biosensor) is now available to screen patients at the point of care and identify individuals with intermediate and severe enzyme deficiency^24^ and is being rolled out in several vivax-endemic countries.^30^ This roll-out allows the wider scale use of tafenoquine and enables the use of higher and more effective doses of primaquine, including broadening the indication for radical cure to those at risk of relapse.

Our study has several limitations. First, the study was powered for the overall sample size but not for site-specific analyses. The recruited sample sizes in Bangladesh and Indonesia were small and the primary endpoint was mainly driven by the Ethiopian site, meaning that generalisation of potential benefits of universal radical cure to different endemic settings should be done with caution. Second, the study was not double-blinded and patients in the intervention arm had more visits than those in the standard care arm, which could have contributed to an observation bias for the tolerability assessment, resulting in an increased reporting of adverse events in the intervention arm. Reassuringly, routine questionnaires on day 2 and day 7 highlighted minimal differences between treatment arms. Third, there are no reliable methods to distinguish whether a patient's recurrent infection arises from recrudescence, re-infection, or relapse.^31^

Universal radical cure confers substantial individual, public health, and operational benefits in regions co-endemic for *P falciparum* and *P vivax,* and this benefit is likely to be greatest in areas of higher endemicity and in countries using artemisinin-based combination therapies, which have low post-treatment prophylaxis, such as artemether–lumefantrine. Many countries endemic for *P vivax* have set ambitious goals to eliminate the parasite within their borders by 2030. Elimination will require wide-scale provision of safer and more effective antimalarial radical cure, with both passive and active case detection. Opportunistically targeting patient populations with a high likelihood of occult hypnozoite carriage, such as patients presenting with *P falciparum* malaria, has the potential to eliminate hidden reservoirs of infection and accelerate elimination.

## Data sharing

The database is closed and the data extracted and stored on WorldWide. Antimalarial Resistance Network (WWARN.org) servers. De-identified individual participant data will be available to applicants who provide a sound proposal to the WWARN Data Access Committee.

## Declaration of interests

KT is funded by a CSL Century fellowship. JAS and RNP are funded by National Health and Medical Research Council Leadership Investigator Grants (1196068 and 2008501). All other authors declare no competing interests.
