# Supplementary material for: Primaquine radical cure in patients with Plasmodium falciparum malaria in areas co-endemic for P falciparum and Plasmodium vivax (PRIMA): a multicentre, open-label, superiority randomised controlled trial
Source: Lancet. 2023 Dec 2;402(10417):2101–10. doi: 10.1016/S0140-6736(23)01553-2 (PMC10714037; doi:10.1016/S0140-6736(23)01553-2)
Supplement: Supplementary appendix [file mmc1.pdf]

THE LANCET

Supplementary appendix

This appendix formed part of the original submission and has been peer reviewed. We post it as supplied by the authors.

Supplement to: Thriemer K, Degaga TS, Christian M, et al. Primaquine radical cure in patients with *Plasmodium falciparum* malaria in areas co-endemic for *P falciparum* and *Plasmodium vivax* (PRIMA): a multicentre, open-label, superiority randomised controlled trial. *Lancet* 2023; published online Nov 15. https://doi.org/10.1016/S0140-6736(23)01553-2.

Supplement to *Primaquine radical cure in patients with Plasmodium falciparum malaria in areas co-endemic for Plasmodium falciparum and vivax (PRIMA) – an open-label randomised controlled trial*

Contents

Table S1: Details of study sites 2

Table S2: List of Ethics Review Boards and Regulatory Agencies 4

Table S3: Dosing charts 6

Table S4: Single dose primaquine in the control arm by country 8

Table S5: Adverse event toxicity table 8

Figure S1: Causal diagram for per protocol analysis 9

Table S6: Protocol deviations and violations 10

Table S7: Recruitment periods by study site 10

Table S8: Number of *P. vivax* recurrences by study site 11

Table S9: Efficacy outcomes by schizontocidal treatment 12

Table S10: Primary and secondary outcomes in the per-protocol analysis 13

Table S11: Parasite and Fever clearance on day 1,2 and 3 13

Table S12a: General safety outcomes, Bangladesh 14

Table S12b: General safety outcomes, Ethiopia 16

Table S12c: General safety outcomes, Indonesia 18

Table S13: Number of adverse events by day during treatment 20

Table S14: Details of all PQ related adverse events 21

Figure S2: Distribution of Haemoglobin (g/dL) by arm 23

Table S15: Details of patients experiencing haematological safety outcomes 24

Table S1: Details of study sites

	Bangladesh	Indonesia	Ethiopia
**Study center**	Alikadam Upazila Complex	Puskesmas (PHC) Mangili<br>Puskesmas Waijelu<br>Puskesmas Tanaraing	Arba Minch General Hospital
**GPS coordinates of study center**	21.652599/ 92.311903	-10.188929/120.675611<br>-10.219395/120.555014<br>-10.000469/ 120.739976	6.025204/ 37.556696
**Altitude above sea level**	111m	80m	1285m
**Treatment policy (including G6PD testing)**[1]	***P. falciparum***: AL+PQ (single dose 0.25 mg/kg)<br>***P. vivax***: CQ+PQ (0.25 mg/kg for 14 days)<br>**G6PD testing** not mentioned in guidelines (last edition 2017), however roll out of quantitative testing using STANDARD G6PD ongoing in a few high-risk areas since 2022.	***P. falciparum***: DHAP+PQ (single dose 0.25 mg/kg)<br>***P. vivax*** DHAP+PQ (0.25 mg/kg for 14 days), 0.5mg/kg over 14 days (for relapse)<br>**G6PD testing** not explicitly mentioned in guidelines (last edition 2020) but required for relapsing cases with higher dose treatment. Testing however not implemented.	***P. falciparum***: AL+PQ (single dose 0.25 mg/kg)<br>***P. vivax:*** CQ+PQ (0.25 mg/kg for 14 days)<br>**G6PD testing** not required as per guidelines (last edition 2018)
**G6PD prevalence and variants**	2-26%[2],<br>Mahidol[3]	8.%[4],<br>Vanua lava, Chatham, Viangchan[5]	Unknown prevalence,<br>likely A- and Mediterranean

---

[1] Sadhewa A, Cassidy-Seyoum S, Acharya S, Devine A, Price RN, Mwaura M, Thriemer K, Ley B. A review of the current status of G6PD deficiency testing to guide radical cure treatment for vivax malaria. *Pathogens* accepted

[2] Ley B, Kibria MG, Khan WA, Auburn S, Phur CS, Jahan N, Johora FT, Thriemer K, Ami JQ, Hossain MA, Price RN, Koepfli C, Alam MS. Wide range of G6PD activities found among ethnic groups of the Chittagong Hill Tracts, Bangladesh. Negl Trop Dis. 2020. https://doi.org/10.1371/journal.pntd.0008697

[3] Ley B, Alam MS, Kibria MG, Marfurt J, Phru CS, Ami JQ, Thriemer K, Auburn S, Jahan N, Johora FT, Hossain MS, Koepfli C, Khan WA, Price RN. Glucose-6-phosphate dehydrogenase activity in individuals with and without malaria: Analysis of clinical trial, cross-sectional and case-control data from Bangladesh. PLoS Med. 2021. doi: 10.1371/journal.pmed.1003576.

[4] Shimizu, H., Tamam, M., Soemantri, A. et al. Glucose-6-phosphate dehydrogenase deficiency and Southeast Asian ovalocytosis in asymptomatic Plasmodium carriers in Sumba island, Indonesia. J Hum Genet 2005. https://doi.org/10.1007/s10038-005-0271-7

[5] Satyagraha AW, Sadhewa A, Baramuli V, Elvira R, Ridenour C, Elyazar I, et al. G6PD Deficiency at Sumba in Eastern Indonesia Is Prevalent, Diverse and Severe: Implications for Primaquine Therapy against Relapsing Vivax Malaria. PLoS Negl Trop Dis 2015. https://doi.org/10.1371/journal.pntd.0003602

**Approx. catchment population of study center**	79,728	32,750	164,529
**Seasonality of malaria**	Year round with peak in May-October	Year round with peak in November-January	Year round with peak in December-February
**P.v./ P.f. ratio**	30:70	15:85	30:70
**Relapse periodicity**	No information	3 months	3-5 months[6]
**Vectors**	*A.maculatus*, *A.jeyporiensis*, *A.nivipes* and *A.vagus*[7,8]	*A.sundaicus*, *A.subpictus*, *A.barbitrosis*[9]	*A.arabiensis*
**Annual Parasite Index (API)**	21.06[10]	Mangili: 6.07<br>Waijelu: 36.98<br>Tanaraing: 13.9	unknown

AL = Artemether-Lumefantrine, CQ= Chloroquine, PQ= Primaquine, DHAP= Dihydroartemisinin–piperaquine, P.v. = Plasmodium vivax, P.f.= Plasmodium falciparum

[6] Abreha T, Hwang J, Thriemer K, et al. Comparison of artemether-lumefantrine and chloroquine with and without primaquine for the treatment of Plasmodium vivax infection in Ethiopia: A randomized controlled trial. PLoS Med. 2017 May 16;14(5):e1002299. doi: 10.1371/journal.pmed.1002299
[7] Alam, M.S., Khan, M.G.M., Chaudhury, N. et al. Prevalence of anopheline species and their Plasmodium infection status in epidemic-prone border areas of Bangladesh. Malar J 9, 15 (2010). https://doi.org/10.1186/1475-2875-9-15
[8] Alam, M.S., Chakma, S., Khan, W.A. et al. Diversity of anopheline species and their Plasmodium infection status in rural Bandarban, Bangladesh. Parasites Vectors 5, 150 (2012). https://doi.org/10.1186/1756-3305-5-150
[9] Kazwaini, Muhammad, and Majematang Mading. "Jenis Dan Status Anopheles Spp. Sebagai Vektor Potensial Malaria Di Pulau Sumba Provinsi Nusa Tenggara Timur." Indonesian Journal of Health Ecology, vol. 14, no. 2, 2015, pp. 96-105.
[10] Based on 2021 NMEP data

Table S2: List of Ethics Review Boards and Regulatory Agencies

Australia	The Human Research Ethics Committee of the Northern Territory Department of<br>Health (HREC)<br>John Mathews Building (Bldg 58)<br>Royal Darwin Hospital Campus, Rock<br>PO Box: 41096, Casuarina NT 0811, Australia<br>Website: www.menzies.edu.au
United Kingdom	The Oxford Tropical Research Ethics Committee (OxTREC)<br>University of Oxford<br>Research Services, University Offices<br>Willington Square, Oxford OX1 2JD<br>Tel: +44 (0) 1865 (2) 82106<br>E-mail: oxtrec@admin.ox.ac.uk<br>Website: www.admin.ox.ac.uk/rso
Bangladesh	Ethical Review Committee (ERC)<br>Dhaka, Bangladesh<br>P.O. Box 128<br>Tel: =8802 9827001 10<br>Fax: +8802 9827075<br>Email: infor@icddrb.org
Indonesia	The Health Research Ethics Committee of the Faculty of Medicine University of<br>Indonesia<br>Cipto Mangunkusumo Hospital<br>Jalan Salemba Raya No. 6, Jakarta Pusat 10430<br>Tel: 021-3157008<br>E-mail: ec_fkui@yahoo.co
	Indonesian Food and Drug Agency (BPOM)<br>Jl, Percetakan Negara No. 23 Jakarta Pusat 10560 Indonesia<br>E-mail: infopom@indo.net.id;<br>Tel: (021) 4244691, 4209221, 4263333, 4244755, 4241781, 4244819<br>Fax: (02) 4245139<br>Website: www.pom.go.id
Ethiopia	The National Research Ethics Review Committee (NRERC)<br>Addis Ababa, Ethiopia<br>PO Box: 2490<br>Tel: +251 114-674-353<br>E-mail: most@ethionet.et<br>Fax: +251 114-660-241<br>Website: www.most.gov.et
	Scientific & Ethical Review Committee (SERC)

	Ethiopian Public Health Institute<br>Addis Ababa, Ethiopia<br>PO Box: 1242/5654<br>E-mail: ephi@ethionet.et<br>Tel: +251 11 2133499, +251 11 2751522<br>Fax: +251 11 2758634<br>Website: www.ephi.gov.et
	The Food Medicine and Health Care Administration and Control Authority<br>(FMHACA)<br>Addis Ababa, Ethiopia<br>Tel: 251-11-552 41 22/552 41 23<br>E-mail: regulatory@fmaca.gov.et<br>Fax: 251-11-552 13 92<br>PO Box: 5681

Table S3: Dosing charts

Schizontocidal treatment for *P. falciparum* patients in Bangladesh and Ethiopia

Artemether-lumefantrine tablet containing 120 mg artemether and 20 mg lumefantrine in a fixed dose.

Body weight (kg)	Day 1		Day 2		Day 3		Colour code
	AM	PM	AM	PM	AM	PM	
5-14	1 tablet	1 tablet	1 tablet	1 tablet	1 tablet	1 tablet	Yellow*
15-24	2 tablets	2 tablets	2 tablets	2 tablets	2 tablets	2 tablets	Blue*
25-34	3 tablets	3 tablets	3 tablets	3 tablets	3 tablets	3 tablets	Brown
>35	4 tablets	4 tablets	4 tablets	4 tablets	4 tablets	4 tablets	Green

* (yellow, blue) - Flavoured paediatric formulation (dispersible tablets) of artemether-lumefantrine (AL) is available for use in young children.

Schizontocidal treatment for *P. falciparum* patients in Indonesia

Body weight (kg)	Daily dose (mg)		Tablet strength and number of tablets per dose
	Piperaquine	Dihydroartemisinin (DHA)	
5 - <7	80	10	½ x 160mg/20mg tablet
7 - <13	160	20	1 x 160mg/20mg tablet
13 - <24	320	40	1 x 320mg/40mg tablet
24 - <36	640	80	2 x 320mg/40mg tablets
36 to <75	960	120	3 x 320mg/40mg tablets
75 to 100	1280	160	4 x 320mg/40mg tablets

Hypnozoitocidal treatment for G6PD normal patients in the high dose PQ7 arm

Primaquine (PQ) (each tablet contains 15 mg PQ) given daily for 7 days with food (daily target dose 1mg per kg body weight):

**Body weight band**	**Body weight (kg)**	**Actual dose given (mg/day)**	**Actual dose given (mg/kg/day)**
A	5 - 22	see suspension dosing chart below	
A	23 - 34	30	0.88 - 1.3
B	35 - 45	45	1 - 1.29
C	≥ 46	60	≤1.33

**Body weight (kg)**	**ml/day**	Two 15mg tablets dissolved in 5ml syrup (1ml=6mg)			
		**Actual dose given (mg/day)**	**Actual dose given (mg/kg/day)**	**Total dose (mg/kg)**	**Total dose (mg)**
5	0.8	4.8	0.96	6.72	33.6
6	1	6	1	7	42
7	1.2	7.2	1.03	7.2	50.4
8	1.3	7.8	0.98	6.83	54.6
9	1.5	9	1	7	63
10	1.7	10.2	1.02	7.14	71.4
11	1.8	10.8	0.98	6.87	75.6
12	2	12	1	7	84
13	2.2	13.2	1.02	7.11	92.4
14	2.3	13.8	0.99	6.9	96.6
15	2.5	15	1	7	105
16	2.5	15	0.94	6.56	105
17	3	18	1.06	7.41	126
18	3	18	1	7	126
19	3	18	0.95	6.63	126
20	3	18	0.9	6.3	126
21	3.5	21	1	7	147
22	3.5	21	0.95	6.68	147

Table S4: Single dose primaquine in the control arm by country

	**Bangladesh**	**Indonesia**	**Ethiopia**
Dosing instruction in national guidelines for transmission blocking single dose primaquine for *P. falciparum* malaria	0.25mg/kg	>6kg-17kg ¼ tablet<br>>17kg-30kg ½ table<br>>30-40 kg ¾ tablet<br>>40kg 1 tablet	19-24kg ¾ tablet<br>25-35 kg 1 tablet<br>36-50kg 1 ½ tablet<br>>50 kg 2 tablet
Tablet strength	15mg	15mg	7.5mg
Manufacturer	JASOPRIM, Jayson pharmaceutical Ltd	Phapros	Remedica

Table S5: Adverse event toxicity table

**Grade**	**Degree of severity**
1	Mild, with no or mild symptoms, in intervention required
2	Moderate, minimal intervention indicated, some limitation of activities
3	Severe but not life-threatening, hospitalization required, limitation of patient's ability to care for him/herself
4	Life-threatening, urgent intervention required
5	Fatal

Figure S1: Causal diagram for per protocol analysis

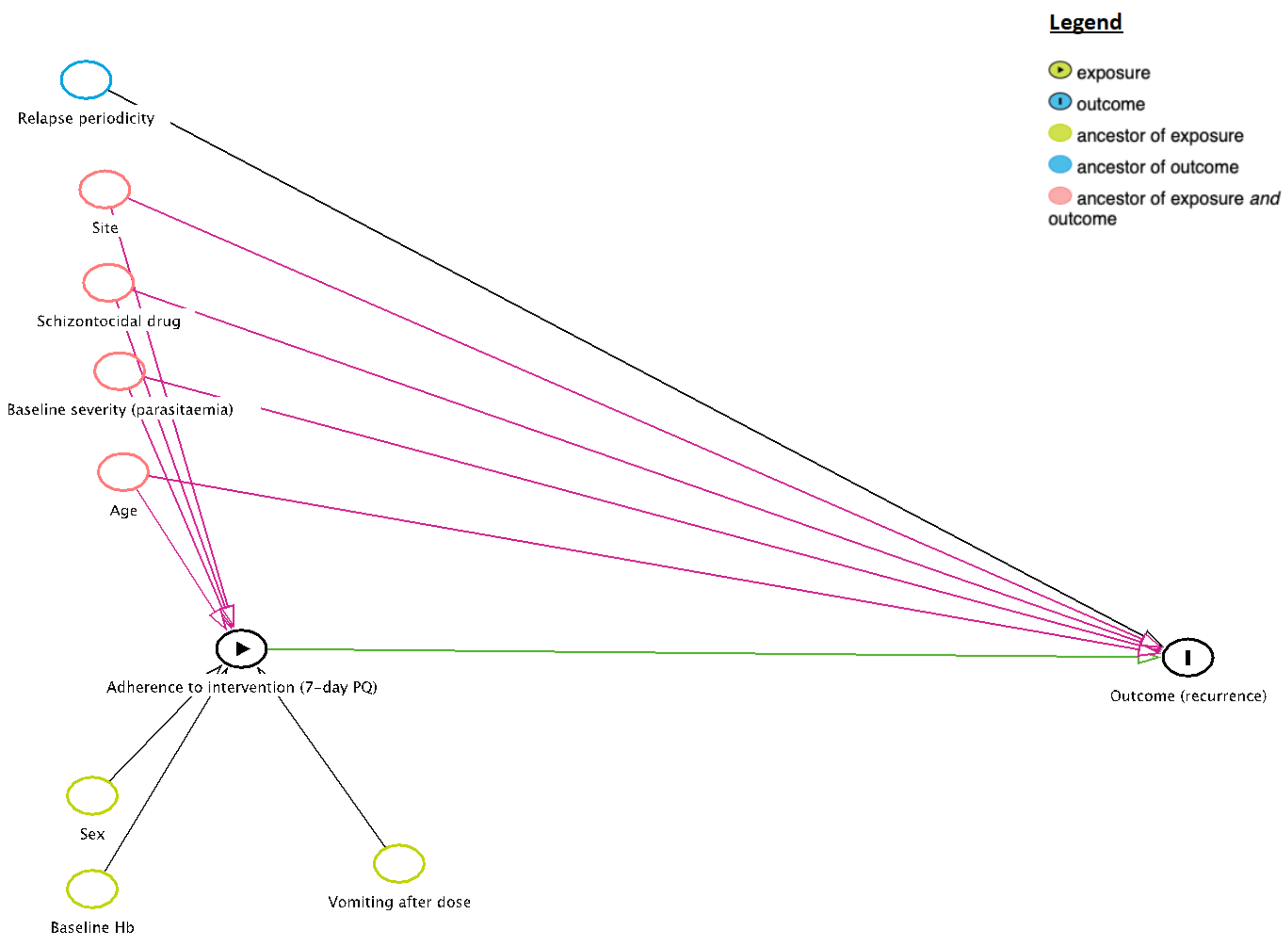

Table S6: Protocol deviations and violations

Site	Finding	Deviation/ violation	Comment
Ethiopia	Recruitment of patient 17 years and 11 months	violation	Protocol violation discovered during monitoring visits. Staff retrained on inclusion and exclusion criteria
	Two patients with PQ administration on day 1 and 2despite evidence of hemoglobinuria (Hillmen score 5)	Deviation from SOP	Discovered during monitoring visit. Staff retrained on SOP
	Three patients with PQ administration on day 4,5 and 1 despite evidence of 25% Hb drop	Deviation from SOP	Discovered during monitoring visit. Staff retrained on SOP
Indonesia	Additional DHP dose given on day 4	Violation	Patient was clinically observed. No adverse events recorded. Study team was retrained
	A randomization envelope was opened despite subject was not eligible due to the manifestation of severe malaria	Violation	Patient was not included in analysis. Study team retrained.
	Study visit was done outside the window period for 6 patients	Deviation	Visit was done outside the window period because subject could not attend the scheduled visit
	End of study medication not provided to 4 patients	Deviation	Re-measurement of G6PD at end of study excluded them from treatment

Table S7: Recruitment periods by study site

	Recruitment start	Recruitment end
Bangladesh	21 August 2019	16 November 2019
Ethiopia	29 January 2021	14 March 2022
Indonesia	1 June 2021	28 December 2021

Table S8: Number of *P. vivax* recurrences by study site

	Treatment arm	
	**High dose PQ7**	**Standard care**
Bangladesh		
Number of patients on day 0	23	24
Number of *P.vivax* recurrences between days 7-63	0	4
Ethiopia		
Number of patients on day 0	175	175
Number of *P.vivax* recurrences between days 7-63	5	19
Indonesia		
Number of patients on day 0	48	50
Number of *P.vivax* recurrences between days 7-63	0	1

Table S9: Efficacy outcomes by schizontocidal treatment

			Incidence risk, % (95%CI)		HR (95%CI)	p value
			**High dose PQ7**	**Standard care**		
**AL**	**Day 63**	All (symptomatic and asymptomatic) *P. vivax* parasitaemia	3.0 (1.3-7.2)	13.2 (9.0,19.3)	0.20 (0.08-0.54)	0.001
		Symptomatic *P. vivax* parasitaemia	0.6 (0.1-3.9)	5.1 (2.6-10.0)	0.12 (0.01-0.92)	0.042
		Any *P. falciparum* malaria	7.3 (4.3-12.2)	6.9 (3.9-12.2)	1.09 (0.49-2.43)	0.835
		P. falciparum gametocytemia	0.0 (0.0-1.9) *	0.6 (0.1-4.4)	-#	-#
	**Day 42**	All (symptomatic and asymptomatic) *P. vivax* malaria	1.2 (0.3-4.8)	10.1 (6.5-15.6)	0.11 (0.02-0.46)	0.003
		Any *P. falciparum* malaria	1.2 (0.3-4.6)	3.5 (1.6-7.7)	0.32 (0.06-1.57)	0.159
	**Day 28**	All (symptomatic and asymptomatic) *P. vivax* malaria	0.6 (0.1-4.5)	5.4 (2.8-10.1)	0.11 (0.01-0.90)	0.039
		Any *P. falciparum* malaria	0.6 (0.1-4.5)	2.4 (0.9-6.3)	0.24 (0.03-2.17)	0.205
**DHAP**	**Day 63**	All (symptomatic and asymptomatic) *P. vivax* parasitaemia	0.0 (0.0-7.7) *	2.2 (0.3-14.7)	-#	-#
		Symptomatic *P. vivax* parasitaemia	0.0 (0.0-7.7) *	0.0 (0.0-7.1) *	-#	-#
		Any *P. falciparum* malaria	8.9 (3.4-22.0)	13.2 (5.6-29.6)	0.83 (0.22-3.08)	0.777
		P. falciparum gametocytemia	0.0 (0.0-7.7) *	2.2 (0.3-14.7)	-#	-#
	**Day 42**	All (symptomatic and asymptomatic) *P. vivax* malaria	0.0 (0.0-7.7) *	0.0 (0.0-7.1) *	-#	-#
		Any *P. falciparum* malaria	5.0 (1.3-18.7)	6.1 (1.6-22.1)	1.06 (0.15-7.50)	0.957
	**Day 28**	All (symptomatic and asymptomatic) *P. vivax* malaria	0.0 (0.0-7.7) *	0.0 (0.0-7.1) *	-#	-#
		Any *P. falciparum* malaria	2.2 (0.3-14.4)	0.0 (0.0-7.1) *	-#	-#

PQ7 – primaquine (7mg/kg total dose given over 7 days); * 95% CI estimated as a proportion of N at risk on day 7 using the binomial exact method; # Could not be estimated due to zero events in the treatment arm.

Table S10: Primary and secondary outcomes in the per-protocol analysis

Outcome	High dose PQ7	Standard care	HR (95% CI)	p-value
Any *P. vivax* parasitaemia to day 63	2.6 (1.1-6.1)	10.2 (6.8-15.1)	0.21 (0.08-0.56)	0.002
Symptomatic *P. vivax* parasitaemia to day 63	0.5 (0.1-3.3)	3.6 (1.7-7.5)	0.12 (0.02-0.99)	0.048
Any *P. falciparum* parasitaemia to day 63	7.0 (4.3-11.4)	8.3 (5.1-13.2)	0.93 (0.46-1.90)	0.849
Any *P. falciparum* gametocytaemia to day 63	0.0 (0.0-1.6) *	1.0 (0.2-3.9)	-[#]	-[#]
Any *P. vivax* parasitaemia to day 42	1.0 (0.3-4.1)	7.2 (4.5-11.5)	0.12 (0.02-0.52)	0.005
Any *P. falciparum* parasitaemia to day 42	1.5 (0.5-4.6)	3.5 (1.7-7.3)	0.44 (0.11-1.72)	0.239
Any *P. vivax* parasitaemia to day 28	0.6 (0.1-3.9)	4.9 (2.6-8.9)	0.10 (0.01-0.83)	0.033
Any *P. falciparum* parasitaemia to day 28	0.6 (0.1-3.9)	2.0 (0.8-5.3)	0.24 (0.03-2.20)	0.208

Models were adjusted for age and baseline parasite density, with stratification by study site. Schizontocidal drug was omitted from models due to high degree of correlation with study site. PQ7 = primaquine (7mg/kg total dose given over 7 days); HR = Hazard Ratio; CI = Confidence Interval. * 95% CI estimated as a proportion of N at risk on day 7 using the binomial exact method; [#] Could not be estimated due to zero events in the study arms.

Table S11: Parasite and Fever clearance on day 1,2 and 3

	High dose PQ7	Standard care	p
Proportion of patients with parasites on day 1	74% (180/242)	77% (191/247)	0.446
Proportion of patients with parasites on day 2	6% (15/241)	7% (18/246)	0.631
Proportion of patients with fever on day 1*	16% (18/114)	9% (10/108)	0.143
Proportion of patients with fever on day 2*	2% (2/114)	2% (2/108)	0.956

The p-value was calculated using a two-sample test of proportions comparing proportions of patients experiencing the outcome in each study arm on each day. *Included only patients febrile at enrolment; PQ7 – primaquine (7mg/kg total dose given over 7 days). The proportion of patients with parasitaemia or febrile in each arm on each day was compared using a two-sample test of proportions.

Table S12a: General safety outcomes, Bangladesh

	High dose PQ7	Standard care
Number of patients randomised (n)	23	24
Adverse events until day 42	1	4
Adverse events (PQ related)*	0	0
Adverse events (PQ un-related)*	1	4
Adverse events until day 7	1	0
Adverse events (PQ related)*	0	0
Adverse events (PQ un-related)*	1	0
Adverse events between day 8 and 42	0	4
Adverse events (PQ related)*	0	0
Adverse events (PQ un-related)*	0	4
Adverse events grade 1 (to day 42)	1	4
Adverse events grade 2 (to day 42)	0	0
Adverse events grade 3 (to day 42)	0	0
Serious adverse events (PQ related)*	0	0
Serious adverse events (PQ un-related)*	1	0
Patients vomiting any medication within 1 hour of administration on a given day, N (%)	0/159	2/87
Number of patients vomiting any PQ dose within 1 hour, N (%)	0/160	1/23
**Symptoms reported on day 2**	**N=23**	**N=23**
Vomiting	4 (17%)	2 (9%)
Headache	14 (61%)	17 (74%)
Nausea	6 (26%)	9 (39%)
Diarrhoea	1 (4%)	0 (0%)
Loss of appetite	20 (87%)	19 (83%)
Abdominal pain	7 (30%)	1 (4%)
Muscle pain	10 (43%)	6 (26%)
Joint pain	10 (43%)	7 (30%)
Fever	13 (57%)	17 (74%)
Dark urine	18 (78%)	20 (87%)
Dizziness	14 (61%)	17 (74%)
Shortness of breath	0 (0%)	1 (5%)[#]
Irritability	13 (57%)	17 (74%)
Jaundice	0 (0%)	0 (0%)[#]
Fatigue	18 (78%)	18 (78%)
Malaise	18 (78%)	19 (83%)

Chills	12 (52%)	16 (70%)
**Symptoms reported on day 7**	**N=8**	**N=17**
Vomiting	0 (0%)	0 (0%)^
Headache	0 (0%)	3 (18%)
Nausea	0 (0%)	1 (6%)
Diarrhoea	0 (0%)	0 (0%)
Loss of appetite	2 (25%)	8 (47%)
Abdominal pain	3 (38%)	0 (0%)
Muscle pain	0 (0%)	1 (6%)
Joint pain	0 (0%)	3 (18%)
Fever	2 (25%)	1 (6%)
Dark urine	6 (75%)	11 (65%)
Dizziness	0 (0%)	2 (12%)
Shortness of breath	0 (0%)	0 (0%)
Irritability	0 (0%)	5 (29%)
Jaundice	1 (12%)	0 (0%)
Fatigue	0 (0%)	1 (6%)
Malaise	0 (0%)	1 (6%)
Chills	0 (0%)	0 (0%)

*Related events include those that are possibly, probably, or definitely related. PQ7 – primaquine (7mg/kg total dose given over 7 days), #Data available for 22 patients from the Standard care arm for shortness of breath and jaundice on day 2, ^Data available for 16 patients from the Standard care arm for vomiting on day 7.

Table S12b: General safety outcomes, Ethiopia

	**High dose PQ7**	**Standard care**
Number of patients randomised (n)	169	165
Adverse events until day 42	41	14
Adverse events (PQ related)*	10	0
Adverse events (PQ un-related)*	31	14
Adverse events until day 7	18	8
Adverse events (PQ related)*	6	0
Adverse events (PQ un-related)*	12	8
Adverse events between day 8 and 42	23	6
Adverse events (PQ related)*	4	0
Adverse events (PQ un-related)*	19	6
Adverse events grade 1 (to day 42)	37	13
Adverse events grade 2 (to day 42)	4	1
Adverse events grade 3 (to day 42)	0	0
Serious adverse events (PQ related)*	0	0
Serious adverse events (PQ un-related)*	0	0
Patients vomiting any medication within 1 hour of administration on a given day, N (%)	0/1201	0/556
Number of patients vomiting any PQ dose within 1 hour, N (%)	0/1200	0/174
**Symptoms reported on day 2**	**N=172**	**N=174**
Vomiting	6 (3%)	7 (4%)
Headache	55 (32%)	77 (44%)
Nausea	9 (5%)	7 (4%)
Diarrhoea	1 (1%)	4 (2%)[#]
Loss of appetite	59 (34%)	64 (37%)
Abdominal pain	3 (2%)	6 (3%)[#]
Muscle pain	70 (41%)	78 (45%)
Joint pain	69 (40%)	80 (46%)
Fever	44 (26%)	53 (30%)
Dark urine	3 (2%)	2 (1%)
Dizziness	50 (29%)	56 (32%)
Shortness of breath	0 (0%)	1 (1%)[#]
Irritability	0 (0%)	0 (0%)
Jaundice	0 (0%)	0 (0%)[#]
Fatigue	69 (40%)	84 (48%)
Malaise	30 (17%)	50 (29%)
Chills	30 (17%)	45 (26%)

**Symptoms reported on day 7**	**N=163**	**N=150**
Vomiting	3 (2%)	0 (0%)
Headache	4 (2%)	4 (3%)
Nausea	4 (2%)	0 (0%)[∀]
Diarrhoea	2 (1%)	0 (0%)
Loss of appetite	3 (2%)[^]	1 (1%)
Abdominal pain	4 (2%)[^]	0 (0%)
Muscle pain	3 (2%)	2 (1%)[∀]
Joint pain	3 (2%)	2 (1%)
Fever	2 (1%)	2 (1%)
Dark urine	0 (0%)	0 (0%)
Dizziness	3 (2%)[^]	2 (1%)
Shortness of breath	0 (0%)[^]	0 (0%)
Irritability	0 (0%)	0 (0%)
Jaundice	0 (0%)	0 (0%)
Fatigue	3 (2%)	2 (1%)
Malaise	2 (1%)	1 (1%)
Chills	1 (1%)	2 (1%)

*Related events include those that are possibly, probably, or definitely related. PQ7 – primaquine (7mg/kg total dose given over 7 days), [#]Data available for 173 patients from the Standard care arm for diarrhoea, abdominal pain, shortness of breath and jaundice on day 2, [^]Data available for 162 patients from the High dose PQ7 arm for loss of appetite, dizziness and shortness of breath on day 7, [∀]Data available for 149 patients from the Standard care arm for nausea and muscle pain on day 7.

Table S12c: General safety outcomes, Indonesia

	High dose PQ7	Standard care
Number of patients randomised (n)	43	49
Adverse events until day 42	55	28
Adverse events (PQ related)*	16	4
Adverse events (PQ un-related)*	39	24
Adverse events until day 7	35	9
Adverse events (PQ related)*	14	3
Adverse events (PQ un-related)*	21	6
Adverse events between day 8 and 42	20	19
Adverse events (PQ related)*	2	1
Adverse events (PQ un-related)*	18	18
Adverse events grade 1 (to day 42)	52	25
Adverse events grade 2 (to day 42)	2	3
Adverse events grade 3 (to day 42)	1	0
Serious adverse events (PQ related)*	0	0
Serious adverse events (PQ un-related)*	1	0
Patients vomiting any medication within 1 hour of administration on a given day, N (%)	1/329	0/148
Number of patients vomiting any PQ dose within 1 h during the 7-day course, N (%)	1/329	0/50
**Symptoms reported on day 2**	**N=40**	**N=40**
Vomiting	5 (12%)	7 (18%)
Headache	27 (68%)	23 (57%)
Nausea	16 (40%)	12 (30%)
Diarrhoea	2 (5%)	3 (8%)
Loss of appetite	9 (22%)	9 (22%)
Abdominal pain	19 (48%)	17 (42%)
Muscle pain	7 (18%)	8 (20%)
Joint pain	5 (12%)	4 (10%)
Fever	15 (38%)	18 (46%)[#]
Dark urine	0 (0%)	0 (0%)
Dizziness	8 (20%)	6 (15%)
Shortness of breath	0 (0%)	1 (2%)
Irritability	0 (0%)	0 (0%)
Jaundice	0 (0%)	0 (0%)
Fatigue	6 (15%)	2 (5%)
Malaise	14 (35%)	15 (38%)
Chills	4 (10%)	7 (18%)

**Symptoms reported on day 7**	**N=22**	**N=20**
Vomiting	1 (5%)	1 (5%)
Headache	5 (23%)	8 (40%)
Nausea	0 (0%)	3 (15%)
Diarrhoea	1 (5%)	1 (5%)
Loss of appetite	2 (9%)	2 (10%)
Abdominal pain	11 (50%)	6 (30%)
Muscle pain	0 (0%)	4 (20%)
Joint pain	0 (0%)	1 (5%)
Fever	4 (18%)	7 (35%)
Dark urine	0 (0%)	0 (0%)
Dizziness	2 (9%)	5 (25%)
Shortness of breath	0 (0%)	0 (0%)
Irritability	0 (0%)	0 (0%)
Jaundice	0 (0%)	0 (0%)
Fatigue	0 (0%)	2 (10%)
Malaise	2 (9%)	5 (25%)
Chills	1 (5%)	3 (15%)

*Related events include those that are possibly, probably, or definitely related. PQ7 – primaquine (7mg/kg total dose given over 7 days), [#]Data available for 39 patients from the Standard care arm for fever on day 2.

Table S13: Number of adverse events by day during treatment

	**High dose PQ7 (n=243)**	**Standard care (n=247)**
Adverse event until day 7	54	17
Adverse events day 0	3	2
Adverse events day 1	7	5
Adverse events day 2	5	5
Adverse events day 3[¥]	6	1
Adverse events day 4[¥]	6	0
Adverse events day 5[¥]	8	0
Adverse events day 6[¥]	9	1
Adverse events day 7	10	3

[¥]reported retrospectively on day 7 for patients in the standard care arm; PQ7 – primaquine (7mg/kg total dose given over 7 days)

Table S14: Details of all PQ related adverse events

ID	Treatment group	Age (years)	Sex	Day of AE occurrence	Type of AE	AE severity	AE relatedness	AE outcome
1	High dose PQ7	8.5	Female	0	acute gastritis	Mild	Probably related	Recovered/resolved
2	Standard care	13.4	Male	0	gastritis	Mild	Probably related	Recovered/resolved
3	Standard care	19	Male	0	gastritis	Mild	Probably related	Recovered/resolved
4	High dose PQ7	13.8	Male	1	gastritis	Mild	Possibly related	Recovering/resolving
5	Standard care	30.6	Male	1	gastritis	Mild	Possibly related	Recovered/resolved
6	High dose PQ7	16.9	Male	2	low back pain	Mild	Possibly related	Recovering/resolving
7	High dose PQ7	22	Female	3	gastritis	Mild	Probably related	Recovered/resolved
8	High dose PQ7	26.9	Male	3	acute gastritis	Mild	Probably related	Recovering/resolving
9	High dose PQ7	18	Female	4	dyspepsia	Mild	Probably related	Recovered/resolved
10	High dose PQ7	19.3	Female	4	epigastric burning	Mild	Probably related	Recovered/resolved
11	High dose PQ7	45	Male	4	gastritis	Mild	Possibly related	Recovered/resolved
12	High dose PQ7	12.2	Female	4	acute gastritis	Moderate	Probably related	Recovered/resolved
13	High dose PQ7	47.8	Female	4	gastritis	Mild	Possibly related	Recovering/resolving
14	High dose PQ7	18	Female	5	epigastric burning	Mild	Possibly related	Recovered/resolved
15	High dose PQ7	12	Female	5	acute upper respiratory tract infection	Mild	Probably related	Recovering/resolving

16	High dose PQ7	16.8	Male	5	gastritis	Mild	Probably related	Recovered/resolved
17	High dose PQ7	18	Male	5	acute gastritis	Severe	Probably related	Recovered/resolved
18	High dose PQ7	16.6	Female	6	light headedness	Mild	Possibly related	Recovering/resolving with sequelae
19	High dose PQ7	15.7	Female	6	acute gastritis	Mild	Probably related	Recovered/resolved
20	High dose PQ7	22	Female	7	gastritis	Mild	Probably related	Recovered/resolved
21	High dose PQ7	19	Male	7	gastritis	Mild	Probably related	Recovered/resolved
22	High dose PQ7	35.4	Female	7	gastritis	Mild	Possibly related	Recovered/resolved
23	High dose PQ7	12.7	Female	7	gastritis	Mild	Possibly related	Recovered/resolved
24	High dose PQ7	21	Female	8	gastritis	Mild	Definitely related	Recovered/resolved
25	High dose PQ7	22	Female	8	gastritis	Mild	Possibly related	Recovered/resolved
26	High dose PQ7	18	Female	9	dyspepsia	Mild	Possibly related	Recovered/resolved
27	Standard care	6.9	Male	9	common cold	Mild	Probably related	Recovering/resolving
28	High dose PQ7	20.9	Female	14	gastritis	Mild	Possibly related	Recovered/resolved
29	High dose PQ7	53.8	Female	28	acute gastritis	Mild	Possibly related	Recovering/resolving with sequelae
30	High dose PQ7	1.7	Female	30	common cold	Mild	Probably related	Recovered/resolved

Patients in the intervention arm were seen more frequently than patients in the control arm (planned follow up visit on each day of treatment in the intervention arm compared to only a day 7 visit in control arm).

Figure S2: Distribution of Haemoglobin (g/dL) by arm

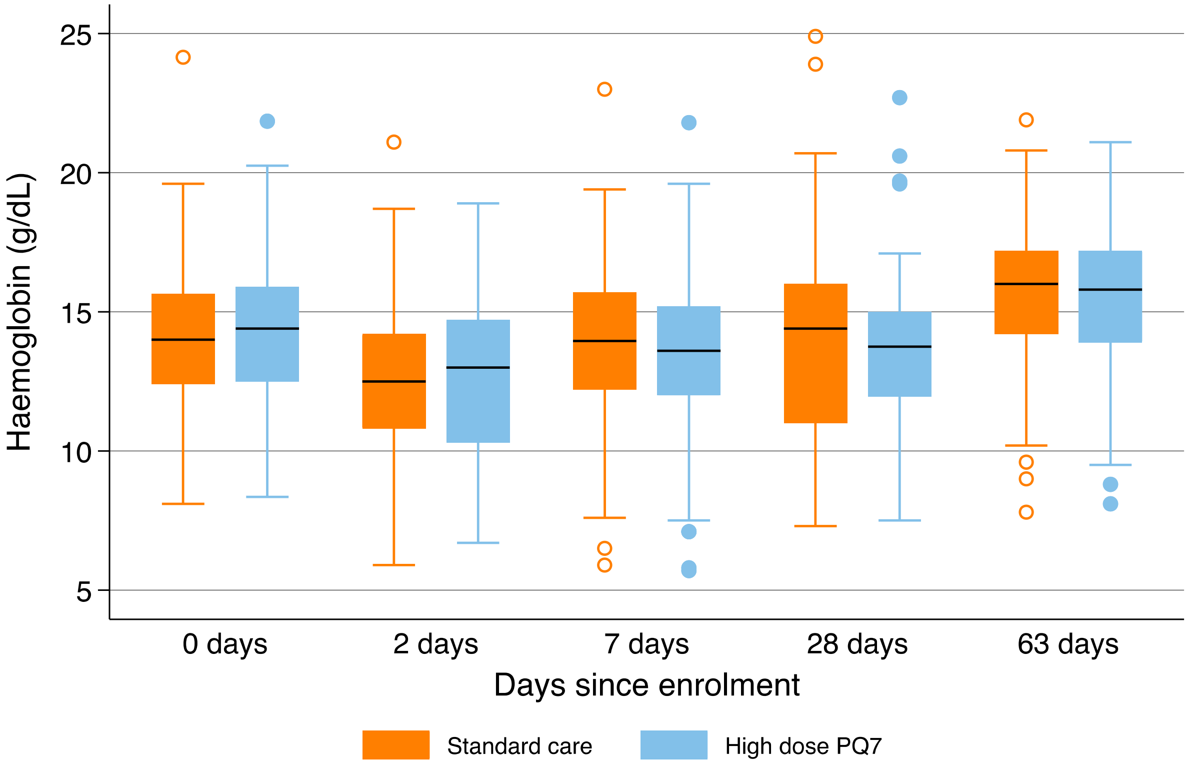

Table S15: Details of patients experiencing haematological safety outcomes

id	Study arm	Study site	Age (years)	Sex	G6PD Day 0 (U/gHb)	Day 0 Hb (g/dl)	Day 2 Hb (g/dl)	Day 7 Hb (g/dl)	Anaemia status day 2	Hb drop of >=25% to <7g/dl day 2	Haemo-globinuria day 2	Anaemia status day 7	Hb drop of >=25% to <7g/dl day 7	Haemo-globinuria day 7	Day of Hb recovery
1	Standard care	Bangladesh	2.5	Fem	12.6	10.4	5.9	9.6	Moderately severe	Yes	Yes	Nil	No	No	21
2	Standard care	Bangladesh	9	Male	8.1	8.4	7.3	10.8	Moderate	No	No	Nil	No	No	7
3	Standard care	Indonesia	6	Male	13.1	8.5	7.7	5.9	Moderate	No	No	Moderately severe	Yes	No	28
4	Standard care	Bangladesh	11	Male	10.2	10	7.7	10	Moderate	No	No	Nil	No	No	7
5	Standard care	Bangladesh	7	Fem	8	9.6	7.8	7.6	Moderate	No	No	Moderate	No	No	14
6	Standard care	Indonesia	14.6	Fem	7.2	8.6	7.8	8.7	Moderate	No	No	Nil	No	No	7
7	Standard care	Bangladesh	12	Male	15.3	10.1	8.9	6.5	Nil	No	No	Moderately severe	Yes	Yes	21
8	Standard care	Bangladesh	50	Fem	16.18	10.8	9.9	7.9	Nil	No	No	Moderate	No	No	63
9	High dose PQ7	Indonesia	1.7	Fem	11.1	9.35	6.7	.	Moderately severe	Yes	No	.	.	.	65
10	High dose PQ7	Indonesia	12.7	Fem	8.95	16.75	7.6	13.5	Moderate	No	No	Nil	No	No	63
11	High dose PQ7	Bangladesh	7	Fem	10	9.9	8.6	7.1	Nil	No	No	Moderate	No	.	36
12	High dose PQ7	Bangladesh	11	Male	9.4	9.2	8.9	5.7	Nil	No	No	Moderately severe	Yes	Yes	28
13	High dose PQ7	Bangladesh	79	Male	14	8.6	9	7.1	Nil	No	No	Moderate	No	.	14
14	High dose PQ7	Bangladesh	42	Fem	8	10.8	9.7	5.8	Nil	No	No	Moderately severe	Yes	Yes	.
15	High dose PQ7	Bangladesh	29	Male	10.9	14.4	13.7	7.5	Nil	No	No	Moderate	No	No	.

PQ7 – primaquine (7mg/kg total dose given over 7 days); Hb – haemoglobin. Anaemia status: Nil: Day 0 Hb>= 8g/dl and stays >= 8/dl, Moderate: Day 0 Hb>=8g/dl and drops to 7-<8g/dl, Moderately severe: Day 0 Hb>=8g/dl and drops to 5-<7g/dl, Severe: Day 0 Hb>=8g/dl and drops to <5g/dl. A total of 4 patients had falls in Hb between day 7 and 28 (3 in the standard care arm and 1 in the high dose PQ7 arm).
